# Activating PIK3CA mutations in adipose-derived stem cells drive mutant-like phenotypes of wild-type cells in macrodactyly

**DOI:** 10.1038/s41419-025-07795-7

**Published:** 2025-07-01

**Authors:** Xiao Zhang, Yating Yin, Zhibo Wang, Yongkang Jiang, Aiping Yu, Xinyi Dai, Xiaoli Wang, Xuesong Guo, Hailei Mao, Bin Wang

**Affiliations:** 1 Department of Plastic and Reconstructive Surgery, Shanghai Key Laboratory of Tissue Engineering, Shanghai Ninth People’s Hospital, Shanghai Jiao Tong University School of Medicine, Shanghai, China; 2 Department of Plastic and Burn Surgery, Children’s Hospital of ShanXi (Women Health Center of ShanXi), Taiyuan, China; 3 Department of Anesthesiology and Critical Care Medicine, Zhongshan Hospital, Fudan University, Shanghai, China

**Keywords:** Cell biology, Diseases

## Abstract

Macrodactyly is a congenital overgrowth disorder characterized by pathological adipose proliferation due to PIK3CA mutations in adipose-derived stem cells (ADSCs). Due to the somatic mosaicism, the affected tissues comprise a mixture of mutant and wild-type cells. However, how PIK3CA mutated ADSCs influence adjacent wild-type cells in macrodactyly remains poorly understood. In this study, we utilized coculture systems to investigate the effects of macrodactylous adipose-derived stem cells (Mac-ADSCs) on normal ADSCs, fibroblasts (FBs), and vascular endothelial cells (VECs). Our study demonstrated that activating PIK3CA mutations in Mac-ADSCs promotes the proliferation, migration, invasion, adipogenesis, and angiogenesis of wild-type ADSCs, FBs and VECs. Furthermore, using RNA sequencing and cytokine arrays, we revealed that these effects are primarily mediated by various secreted paracrine cytokines. These findings demonstrated that activating PIK3CA mutation alters the paracrine characteristics of Mac-ADSCs and reshapes the microenvironment of macrodactyly, driving adjacent wild-type cells to exhibit mutant-like phenotypes. Targeting PIK3CA with BYL-719 could influence the progression of macrodactyly by inhibiting the paracrine signaling of Mac-ADSCs.

## Introduction

Macrodactyly is a rare congenital condition with an incidence of approximately 1 in 100,000 live births [1]. Histologically, macrodactyly is characterized by complex structural lesions with enlarged adipose tissue, accompanied by uneven and disorganized extracellular matrix [2, 3]. These lesions are thought to progress in severity with time, and are associated with limb dysfunction [1]. Treatment for macrodactyly, especially for complex malformations that include grossly deformed and giant distal limbs, is challenging. Severe hypertrophy of the extremities often results in significant inconveniences for patients and their families. Therefore, it is crucial to elucidate the molecular mechanisms underlying this condition.

Somatic mosaic mutations of phosphatidylinositol-4,5-bisphosphate 3-kinase, catalytic subunit alpha (PIK3CA) gene in limb tissues are the main cause of macrodactyly [4]. The Histidine 1047 to Arginine (H1047R) mutation is the most common PIK3CA mutation. This mutation occurs within the kinase domain of PIK3CA, bypassing the requirement for association with Ras and thereby increasing the accessibility of the kinase domain to the membrane-bound phosphatidylinositol 4,5-bisphosphate (PIP2) substrate [5]. PIK3CA with activating mutations primarily recruits specific proteins, including serine/threonine kinases, guanine nucleotide exchange factors (GEFs) and GTPase-activating proteins (GAPs) [6], to membrane signaling complexes to initiate distinct enzymatic or signaling functions. In response to PI3KCA activation, multiple downstream pathways are activated, leading to changes in cell proliferation, metabolism, and cytokine secretion [7]. During development, somatic activating mutations caused by PIK3CA can lead to a range of diseases, including tissue hyperplasia, vascular malformations, and brain developmental abnormalities [8]. Owing to the clinical overlap of these disorders caused by PIK3CA mutations, they are classified under the PIK3CA-related overgrowth spectrum (PROS) [9].

The excessive adipose tissue accumulation is the most prominent characteristic of macrodactylous hypertrophy. Adipose tissue mass is regulated by adipose-derived mesenchymal stem cells (ADSCs), which can differentiate into mature adipocytes [10]. These cells exhibit the capacity for self-renewal and differentiation into multiple cell types, including mesenchymal and nonmesenchymal lineages [11]. Our previous work demonstrated excessive adipose accumulation and increased differentiation of ADSCs in macrodactyly caused by PIK3CA mutation [12, 13]. However, the impact of these mutated ADSCs on surrounding tissue remains to be fully elucidated.

Recent studies have increasingly emphasized the pivotal paracrine role of ADSCs, in addition to their intrinsic differentiation potential [14]. Paracrine signaling refers to the process by which cells communicate with neighboring cells by secreting diffusible signaling molecules. In the process of tissue growth and repair, ADSCs secrete angiogenic cytokines, such as vascular endothelial growth factor (VEGF) and angiogenin, as well as antiapoptotic cytokines, such as insulin-like growth factor-1 (IGF-1), to promote angiogenesis and facilitate tissue regeneration [15]. Moreover, the importance of paracrine signaling has been illustrated in the context of PIK3CA mutations. The PIK3CA^H1047R^ mammary duct contributes to vascular dysfunction via the paracrine signaling of IL-6 [16]. Similarly, PIK3CA-mutated colorectal cancer cells can induce the malignant transformation of intestinal epithelial cells through paracrine exosomal arachidonic acid (AA)-mediated H3K4 trimethylation [17]. Recent multi-omics analysis of gastric adenocarcinoma has also identified an ‘Inflamed’ tumor subgroup with PIK3CA mutations showing distinct immune signaling properties in the tumor microenvironment [18]. In macrodactyly, the affected tissues comprise a mixture of mutant and wild-type cells due to somatic mosaicism. Studies have shown that the average variant allele frequency of PIK3CA is only 21% in isolated macrodactylous tissues [4], indicating that wild-type cells constitute the majority of the lesion. This unique cellular composition suggests that the extensive tissue overgrowth and disproportionate tissue expansion in macrodactyly cannot be explained solely by the autonomous effects of mutant cells. In CLOVES syndrome, Kurek et al. hypothesized that the malformations are the result of paracrine signaling from mutant to wild-type cells [8]. However, the precise mechanisms of this paracrine signaling in macrodactyly remain unexplored.

Given the importance of paracrine signaling in the context of PIK3CA mutations and ADSCs, exploring the interplay between PIK3CA-mutated ADSCs and the cellular environment in influencing the progression of macrodactyly is crucial. In this study, we explored the influence and underlying mechanisms of PIK3CA mutation in ADSCs on adjacent cellular environments. We cocultured Mac-ADSCs with wild-type ADSCs, fibroblasts (FBs), and vascular endothelial cells (VECs) and examined changes in cellular proliferation, migration, invasion, adipogenesis, and angiogenesis. Meanwhile, we identified differentially secreted factors and signaling pathway potentially involved in these interactions. By elucidating the mechanisms of intercellular communication, we hope to provide novel insights into the progression of macrodactyly and identify potential therapeutic targets for its management.

## Methods

### Donor specification and sample collection

This study was conducted in accordance with the guidelines of the Institutional Review Board of Shanghai Ninth People’s Hospital and was approved under the protocol number SH9H-2022-T357-1. Written informed consent was obtained from patients or their legal guardians. Subcutaneous fat tissue samples were collected from macrodactyly and polydactyly patients (under two years old) who underwent debulking or resection surgeries. The detailed clinical information is listed in Table S1.

### Cell preparation

The isolation of ADSCs was performed as previously described [13]. Briefly, Mac-ADSCs were isolated from macrodactylous tissue, whereas Pol-ADSCs were derived from the digits of polydactyly patients. Adipose tissue samples (approximately 1 cm^3^ in size) were obtained under sterile conditions and digested with 0.15% (w/v) collagenase IV (NB4; Serva) for 2 h, followed by centrifugation for 5 min at 1500 rpm to obtain ADSCs. The cells were cultured in low-glucose Dulbecco’s modified Eagle’s medium (DMEM) supplemented with 10% fetal bovine serum and 100 μg/mL penicillin-streptomycin at 37 °C in a 5% CO_2_ incubator until they reached 80–90% confluence. Cells from passage 3 to passage 5 were used for all experiments. FBs and VECs were purchased from iCell (Shanghai, China).

### DNA isolation and sequencing

Genomic DNA extraction and amplification were performed as previously described [12]. Amplified PCR products were analyzed at GENEWIZ (Suzhou, China) for Sanger sequencing.

### Flow cytometric assay

Analysis of the surface markers of ADSCs and flow cytometry were performed as previously described [13]. The primary antibodies used are listed in Table S2. The flow cytometric gating strategy was described in Figure S1.

### Western blotting

For protein extraction, the cells were lysed with RIPA lysis buffer supplemented with phosphatase (EpiZyme, GRF102) and protease inhibitors (EpiZyme, GRF101). The protein concentration was determined with a BCA protein assay kit (Beyotime). The protein was subsequently resolved by sodium dodecyl sulfate-polyacrylamide gel electrophoresis before being transferred onto polyvinylidene difluoride membranes. After incubation with primary antibodies and peroxidase-conjugated secondary antibodies, the blots were detected via enhanced chemiluminescence (ECL) reagent (Thermo Fisher Scientific) on an iBright CL1500 imaging system (Invitrogen). The antibodies used are listed in Table S2. The original unmodified scans of western blots are provided in the Source Data file.

### Coculture system establishment

Coculture systems can be divided into two categories: conditioned medium (CM) systems and Transwell systems. For CM preparation, ADSCs (1 × 10^5^/well) were plated on 6-well plates and cultured overnight. On the next day, the medium was replaced with serum-free DMEM, and the supernatant was harvested after 48 h. The supernatant was then supplemented with an equal volume of fresh culture medium to prepare CM. The establishment of the Transwell system was performed as previously described [19]. Briefly, ADSCs were seeded on the upper chamber of a 0.4 μm polyester membrane (Corning) and wild-type cells were plated below. After 48 h, the cells in the lower chamber were collected and used for subsequent studies.

Neutralization of cytokines in CM was performed via the use of neutralizing antibodies. Specifically, neutralizing antibody or IgG control antibody was added to the supernatant of Mac-ADSCs, which was subsequently incubated at 37 °C for 1 h. The treated CM was then supplemented with an equal volume of fresh culture medium and used for neutralization assays. The antibodies used are listed in Table S2.

### Proliferation assays

Cell proliferation was measured via a Cell Counting Kit-8 assay (Dojindo Laboratories) following the manufacturer’s instructions. Briefly, cells (2 × 10^3^/well) were seeded in 96-well plates. For ADSCs and FBs, the medium was replaced every 48 h and absorbance measurements were taken on days 1, 3, 5, and 7 to assess cell proliferation over the course of one week. For VECs, the medium was replaced daily, and absorbance measurements were taken on days 1, 2, 3 and 4.

### Adipogenic differentiation

Adipogenic differentiation was performed as described previously [13]. Briefly, adipogenic induction media (CM with 10 µg/mL insulin, 0.5 mM isobutyl-1-methylxanthine, 1 µg/mL dexamethasone and 0.2 mM indomethacin) was used to assay the adipogenic differentiation capability of ADSCs. The medium was changed every 3 days. After 12 days of culture, Oil Red O staining (Sigma) was performed according to the manufacturer’s instructions.

### RNA extraction and qRT-PCR

Total RNA was extracted via an EZ-press RNA Purification Kit (EZBioscience). Reverse transcription and qRT-PCR were performed via a Color Reverse Transcription Kit (EZBioscience) and 2× Color SYBR Green qPCR Master Mix (EZBioscience) according to the manufacturer’s instructions. Gene expression levels were relatively quantified with the 2^−ΔΔCT^ method. The primer sequences are listed in Table S3.

### Migration and invasion assays

Scratch assays were used to examine the cell migratory capacity. Briefly, 2 × 10^5^ cells were seeded in six-well plates. After reaching 90-100% confluence, the cells were scratched with a sterile 200 μL pipette tip. The cells were then subjected to different treatment conditions and monitored at the appropriate times. Transwell assays were used to examine both cell migration and invasion. Briefly, the cells were serum-starved for 24 h and then seeded in the upper chamber of an 8.0 μm pore size insert at the appropriate density. For evaluation of invasion, 60 μL of diluted Matrigel matrix (Corning, 356234) was added to the center of each Transwell insert. In the lower chamber, both Fot-ADSCs and Mac-ADSCs were seeded at a density of 5 × 10^4^/well, and complete DMEM was used as a negative control. After 24 h of incubation, the cells on the lower surface of the membrane were stained with 0.5% crystal violet and observed with a microscope. All quantitative images were obtained via ImageJ software.

### Angiogenesis assays

The proangiogenic capacity of Mac-ADSCs was assessed via an in vitro Matrigel angiogenesis assay. Briefly, VECs (1 × 10^5^/well) were seeded in 24-well plates coated with Matrigel matrix (Corning, 354248) after treatment with the indicated medium for 24 h. Tube formation was visualized with microscopy after incubation for 2 to 6 h. Angiogenic capacity was evaluated by ImageJ software.

### Bioinformatics and cytokine array analysis

The transcriptomic profiles were obtained from GSE151840. Differential gene expression analysis was conducted via the R package “DESeq2”. Significant differential expression was defined as padj ≤ 0.05, |log2FoldChange | ≥ 1, and BaseMean ≥ 100. Genes encoding secretory proteins were obtained from the Human Protein Atlas database (https://www.proteinatlas.org/). GO and KEGG enrichment analyses were performed via the R package “clusterProfiler”. The comparison of cytokines secreted by ADSCs was performed via the Human Cytokine Antibody Array C1000 (Raybio) according to the manufacturer’s instructions. Relative cytokine levels were quantified via ImageJ software. Significant differentially secreted proteins were defined as those exhibiting grayscale value changes greater than 1.3 or less than 0.7.

### Lentiviral shRNA production and transduction

Lentivirus production and transient transduction were performed as described previously [12]. Briefly, ADSCs (2 × 10^5^) were seeded into 6-well plates and infected with virus overnight. After being incubated in regular culture medium for 48 h, 2 μg/mL puromycin was added, and the cells were cultured for 3 days. Transfection efficacies were determined by fluorescence, qRT-PCR, and western blotting.

### Statistical analysis

The data are shown as the mean ± standard deviations (SD) from three independent experiments. Homogeneity of variance was tested before statistical analysis. Unpaired *t* tests or one-way ANOVA with Tukey post hoc tests were used to evaluate differences between experimental groups. *p* < 0.05 was considered statistically significant (*p < 0.05, **p < 0.01, ***p < 0.001, and ****p < 0.0001). The statistical analysis was performed using Prism software (version 10.0.2).

## Results

### PIK3CA gain-of-function mutation in Mac-ADSCs

**Fig. 1:**
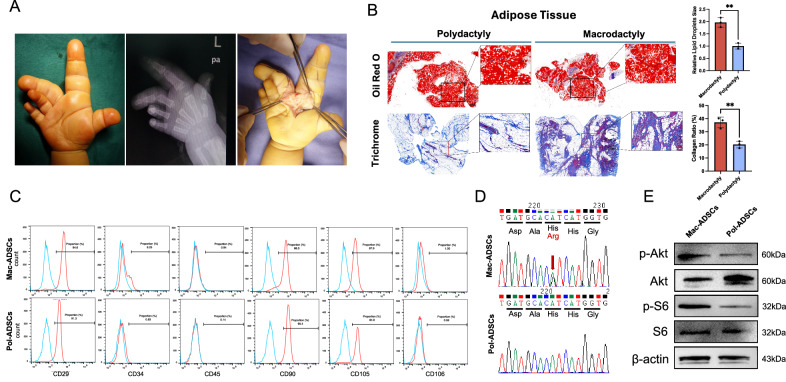
Excessive adipose tissue accumulation and PIK3CA gain-of-function of ADSCs in macrodactyly. **A** Gross appearance and radiograph showed diffuse tissue hyperplasia in macrodactyly. **B** Oil Red O and Masson’s trichrome staining revealing diffuse enlargement of adipocytes and localized collagen deposition of adipose tissue in macrodactyly compared with polydactyly. **C** Flow cytometric analysis of ADSC-specific markers (CD29, CD34, CD45, CD90, CD105, CD106) in Mac-ADSCs and Pol-ADSCs. **D** Sanger sequencing identifying PIK3CA mutations in Mac-ADSCs but not in Pol-ADSCs. **E** Western blot showing activation of the PI3K-AKT pathway in Mac-ADSCs. Data are presented as the mean ± SD from three independent experiments. *p < 0.05, **p < 0.01 by unpaired *t* test.

One of the characteristic manifestations of macrodactyly is the overgrowth of extremities, with marked adipose tissue accumulation in the affected area (Fig. 1A). Oil red O and Masson’s trichrome staining revealed diffuse enlargement of the lipid droplets and localized fiber hyperplasia in macrodactyly samples (Fig. 1B). Given the potent paracrine effects of mesenchymal stem cells, we hypothesized that these pathological changes are induced by the influence of mutated ADSCs on surrounding cells. Mesenchymal stem cells are characterized by the expression of specific surface markers [20]. Flow cytometric analysis revealed that both Mac-ADSCs and Pol-ADSCs were positive for CD29, CD90, and CD105, and negative for CD34, CD45, and CD106 (Fig. 1C). Using Sanger sequencing, we identified a mosaic mutation in PIK3CA (c.3140 A > G, p.H1047R) in Mac-ADSCs (Fig. 1D). Western blot analysis further revealed elevated levels of phosphorylated AKT and S6 in Mac-ADSCs, indicating activation of the PI3K/AKT pathway (Fig. 1E).

### Mac-ADSCs altered the biological behavior of wild-type cells

**Fig. 2:**
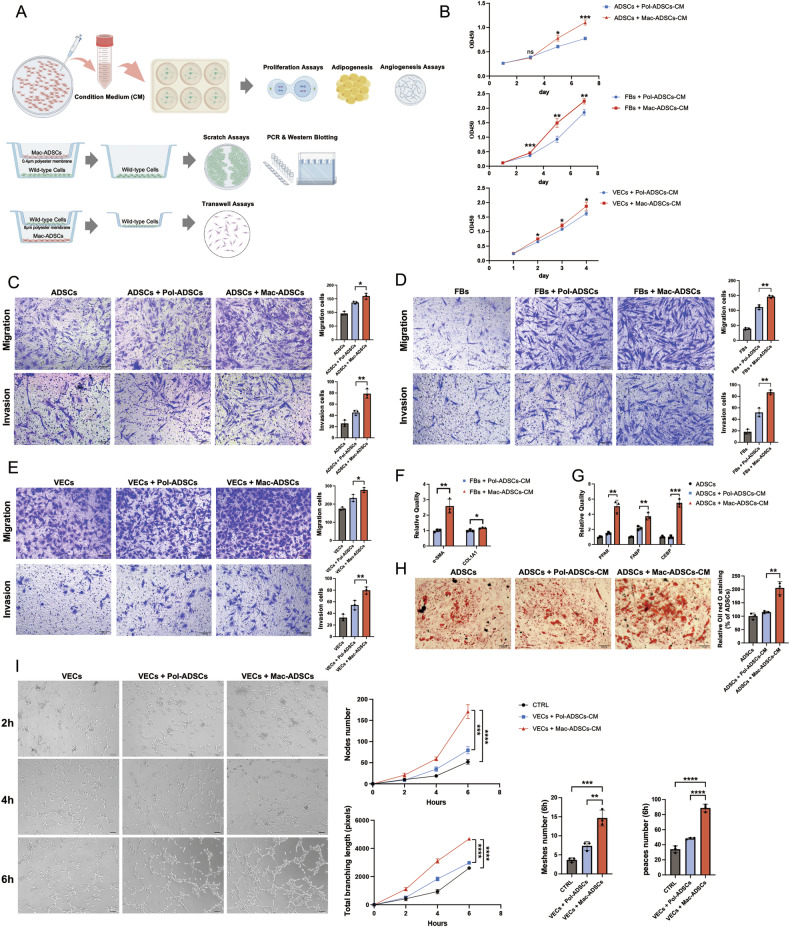
Mac-ADSCs enhance proliferation, migration, invasion and functional changes in wild-type cells. **A** Schematic of the coculture system used for functional assays. The figure was created with Figdraw (https://www.figdraw.com/) and is permitted for publication. **B** Effect of Mac-ADSCs conditioned medium (CM) on proliferation of ADSCs, FBs and VECs assessed by CCK-8 assay. **C**–**E** Migration and invasion capacities of (**C**) ADSCs, (**D**) FBs, and (**E**) VECs evaluated by Transwell assays. **F** mRNA levels of collagen markers in FBs when cocultured with different ADSCs. **G** mRNA levels of adipogenesis markers in ADSCs after 3 days of adipogenic induction. **H** Oil Red O staining of ADSCs after 12 days of adipogenic induction. **I** Representative images of angiogenic tube formation by VECs cocultured with different ADSCs. Scale bars: 100 μm. Data are presented as mean ± SD from three independent experiments. ns, not significant, *p < 0.05, **p < 0.01, ***p < 0.001, ****p < 0.0001 by ANOVA analysis.

To investigate the potential influence of Mac-ADSCs on adjacent normal cells, we utilized several coculture models (Fig. 2A). CCK-8 assays demonstrated that compared with Pol-ADSCs-CM, Mac-ADSCs-CM significantly increased the proliferation of wild-type ADSCs, FBs, and VECs (Fig. 2B). Transwell assays demonstrated that Mac-ADSCs effectively promoted the migration and invasion capabilities of ADSCs, FBs, and VECs (Fig. 2C–E). A parallel scratch assay also revealed a similar trend of increased migration (Figure S2A–C).

In addition to their effects on proliferation and migration, we investigated whether Mac-ADSCs affect adipogenesis and fiber expansion, two major characteristics of macrodactyly. COL1A1 and α-SMA were elevated in FBs when cocultured with Mac-ADSCs, suggesting an increased ability for collagen synthesis (Fig. 2F). The mRNA expression of adipogenic markers (PPAR, FABP, and CEBP) and Oil Red O staining indicated increased adipogenic potential in the Mac-ADSCs-CM group (Fig. 2G, H). Furthermore, angiogenesis assays revealed that Mac-ADSCs promoted angiogenic properties, including a significant increase in the number of nodes, meshes, peaces as well as total branching length (Fig. 2I). Collectively, these results indicate that Mac-ADSCs significantly influence the biological functions of surrounding normal cells and create a unique microenvironment, which may influence the progression and characteristic features of macrodactyly.

### Mac-ADSCs affected surrounding cells via paracrine mechanisms

**Fig. 3:**
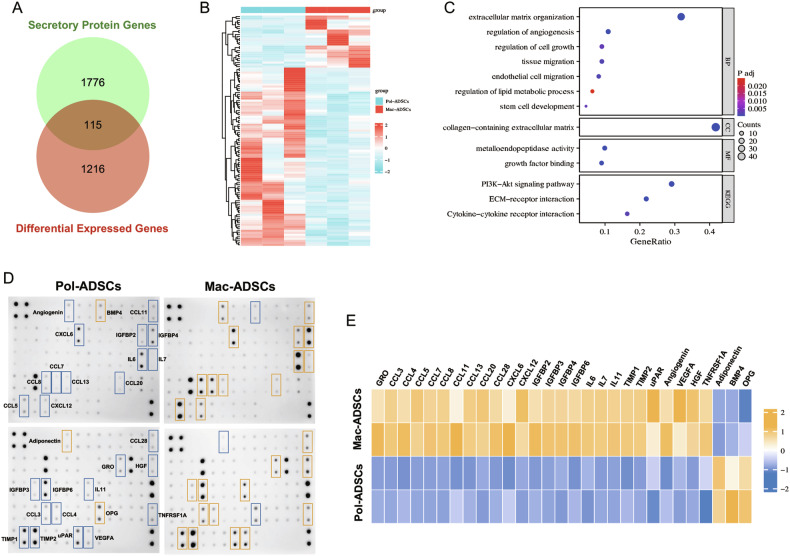
Mac-ADSCs influence wild-type cells through paracrine signaling. **A** Venn diagram showing overlap between the differentially expressed genes (DEGs) and the genes encoding secretory proteins in Mac-ADSCs versus Pol-ADSCs. **B** Heat map of DEGs encoding secretory proteins, with color-coded expression levels. **C** GO/KEGG pathway analysis of DEGs that encode secretory proteins. **D** Human cytokine antibody arrays detecting cytokines in the supernatant of Pol-ADSCs (left) and Mac-ADSCs (right). Boxes highlight cytokines with significant differential expression. **E** Heat map showing the intensities of the dots representing the differentially expressed cytokines (data presented as grayscale values in each group).

Based on our findings that Mac-ADSCs could affect wild-type cells, we sought to investigate the underlying mechanisms mediating these effects. Studies have shown that ADSCs exert their functional benefits through paracrine effects [20, 21]. Therefore, we examined RNA-sequencing data for secretory protein genes. Among the differentially expressed genes, 115 encoded secretory proteins (Fig. 3A, B). The cluster analysis of these genes was performed to identify the functions and pathways involved. As shown in Fig. 3C, the most common GO terms were related to ‘extracellular matrix’, ‘cell growth’, ‘angiogenesis’, ‘tissue migration’, ‘lipid metabolism’, and ‘stem cell development’. The PI3K-AKT signaling pathway was identified as the top-scoring category in the KEGG annotations.

To further investigate the alterations in the paracrine capabilities of PIK3CA-mutant ADSCs, we performed cytokine array analysis on the supernatants of Mac-ADSCs and Pol-ADSCs (Fig. 3D). The grayscale intensities of the cytokines are summarized in Table S4. Cytokine array analysis confirmed the differential secretion of several factors. As shown in Fig. 3E, the differentially expressed cytokines included molecules from the C-C motif ligand (CCL) and C-X-C motif ligand (CXCL) chemokine families, the insulin-like growth factor binding protein (IGFBP) family, the tissue inhibitor of metalloproteinases (TIMP) family, and the interleukin (IL) family. Additionally, certain molecules directly involved in cell migration, angiogenesis, and lipid metabolism also undergo alterations. These results suggest that Mac-ADSCs secrete cytokines that modulate the behavior of surrounding wild-type cells via paracrine mechanisms.

To further explore the specific roles of these secreted cytokines in mediating the paracrine effects of Mac-ADSCs, we employed neutralizing antibodies against IL-6, IL-11, HGF, and VEGFA. These four cytokines were predicted to influence a range of cellular functions relevant to our study. Notably, neutralization of IL-6 resulted in only a minor reduction in the proliferation of these three cell types (Figure S3A) and did not significantly impact their migration (Figure S3B–D). However, we found that IL-6 neutralization significantly increased the adipogenic capacity of wild-type ADSCs when cocultured with Mac-ADSCs (Figure S3E), while it did not affect the angiogenic capacity of VECs (Figure S3F). In contrast, neutralization of IL-11 predominantly inhibited the proliferation and migration of FBs, while showing limited effects on the proliferation and migration of ADSCs and VECs. (Figure S4A–D). Moreover, IL-11 neutralization enhanced the adipogenic differentiation of wild-type ADSCs (Figure S4E) and slightly reduced the pro-angiogenic effect of VECs (Figure S4F) when cocultured with Mac-ADSCs. Furthermore, we investigated the effects of neutralizing antibodies against HGF and VEGFA. As a well-known angiogenic factor, HGF neutralization significantly inhibited the proliferation and migration of VECs, while its effects on ADSCs and FBs were relatively modest, showing only minor increases in the proliferation and migration (Figure S5A–D). Notably, neutralization of HGF suppressed the expression of the key adipogenic gene PPAR in ADSCs, indicating its involvement in Mac-ADSC-mediated adipogenesis (Figure S5E). Meanwhile, it significantly inhibited the angiogenic capacity of VECs (Figure S5F). Additionally, VEGFA neutralization demonstrated the most comprehensive effects among all tested cytokines. Anti-VEGFA antibodies significantly inhibited the proliferation and migration of all three cell types (Figure S6A–D). The adipogenic differentiation of wild-type ADSCs and tube formation ability of VECs were also substantially impaired in the presence of VEGFA neutralizing antibodies (Figure S6E, F).

### PIK3CA-knockdown impaired the paracrine effects of Mac-ADSCs

**Fig. 4:**
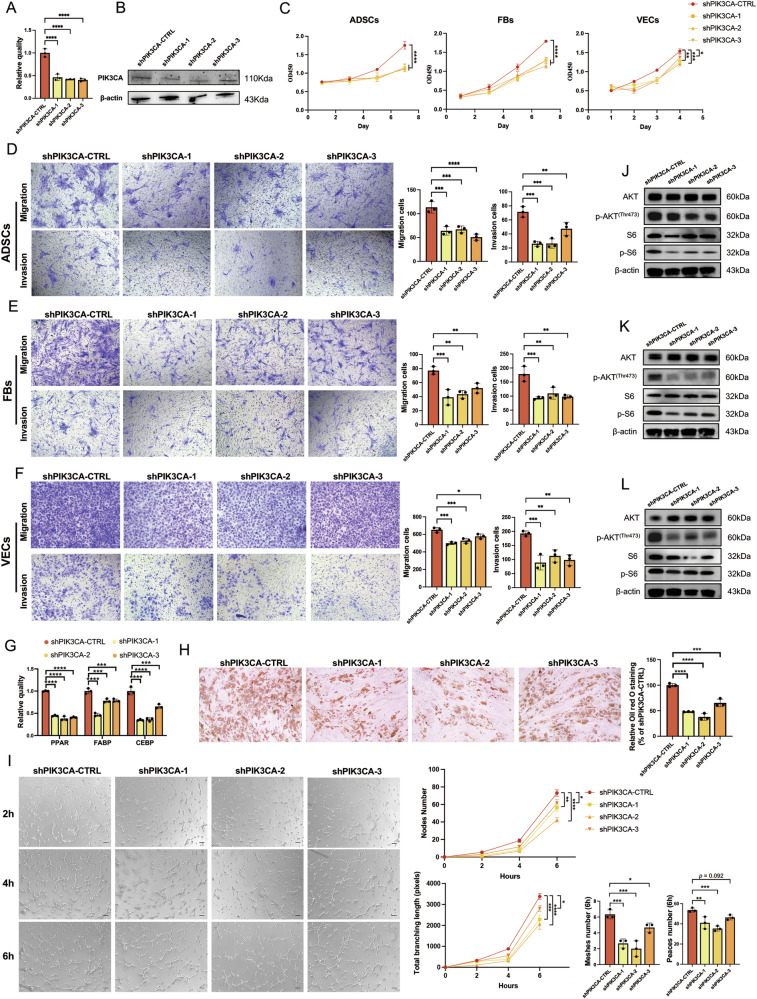
Knockdown of PIK3CA attenuates the paracrine effects of Mac-ADSCs. (**A**) mRNA and (**B**) protein expression levels of PIK3CA in Mac-ADSCs after shRNA transfection. **C** Proliferation of ADSCs, FBs, and VECs when cocultured with shRNA-transfected Mac-ADSCs. **D**–**F** Migration and invasion capacities of (**D**) ADSCs, (**E**) FBs, and (**F**) VECs when cocultured with shRNA-transfected Mac-ADSCs. **G**, **H** Adipogenic differentiation potential of wild-type ADSCs evaluated by (**G**) mRNA expression levels of adipogenic markers and (**H**) Oil Red O staining after coculturing with shRNA-transfected Mac-ADSCs. **I** Tube formation capacity of VECs when cocultured with shRNA-transfected Mac-ADSCs. **J**–**L** Activation status of the PI3K-AKT signaling pathway in (**J**) ADSCs, (**K**) FBs, and (**L**) VECs when cocultured with shRNA-transfected Mac-ADSCs. Scale bars: 100 μm. Data are presented as the mean ± SD from three independent experiments. *p < 0.05, **p < 0.01, ***p < 0.001, ****p < 0.0001 by ANOVA analysis.

To determine whether the paracrine effects of Mac-ADSCs are dependent on PIK3CA activation, we used lentivirus-mediated transduction to knock down PIK3CA (shPIK3CA) in Mac-ADSCs. Effective transduction was confirmed by fluorescence microscopy at a multiplicity of infection (MOI) of 20 (Figure S7). RT-qPCR and western blot analyses demonstrated that shRNAs effectively suppressed PIK3CA expression at both the mRNA and protein levels (Fig. 4A, B), and further revealed that PIK3CA knockdown inhibited its downstream effectors AKT and S6 (Figure S8A). Growth curve analyses revealed that compared with that from control Mac-ADSCs, CM from PIK3CA-knockdown Mac-ADSCs significantly reduced the proliferation of wild-type ADSCs, FBs, and VECs (Fig. 4C and Figure S8B). The paracrine effects on cell movement were also diminished, with reduced migratory and invasive capacities of cocultured cells (Fig. 4D–F and Figure S8C). The adipogenic capacity was evaluated by adipogenesis-related gene expression and Oil Red O staining. As shown in Fig. 4G and Fig. 4H and Figure S8D, the adipogenic potential of wild-type ADSCs was significantly impaired when they were cocultured with PIK3CA-knockdown Mac-ADSCs. Additionally, the capacity of VECs to form vascular structures was inhibited when cocultured with PIK3CA gene-silenced Mac-ADSCs (Fig. 4I and Figure S8E). Interestingly, we found PIK3CA-knockdown Mac-ADSCs exhibited even weaker paracrine effects on wild-type cells compared to Pol-ADSCs. This pronounced reduction in paracrine function may be attributed to the simultaneous suppression of both the basal expression and phosphorylation levels of PIK3CA downstream effectors, resulting in a more comprehensive inhibition of the signaling pathway than is observed in Pol-ADSCs.

Transcriptomic and cytokine array analyses suggested that the cytokines secreted by Mac-ADSCs may activate the PI3K-AKT signaling pathway in surrounding cells. To further investigate the association between this activation and PIK3CA mutation, we evaluated the protein levels in wild-type cells after coculture with PIK3CA-silenced Mac-ADSCs. The results revealed a significant decrease in the phosphorylation levels of AKT and S6 in ADSCs (Fig. 4J), FBs (Fig. 4K), and VECs (Fig. 4L), indicating that PIK3CA activation in Mac-ADSCs plays a critical role in mediating their paracrine effects.

### BYL-719 suppressed the paracrine effects of Mac-ADSCs

**Fig. 5:**
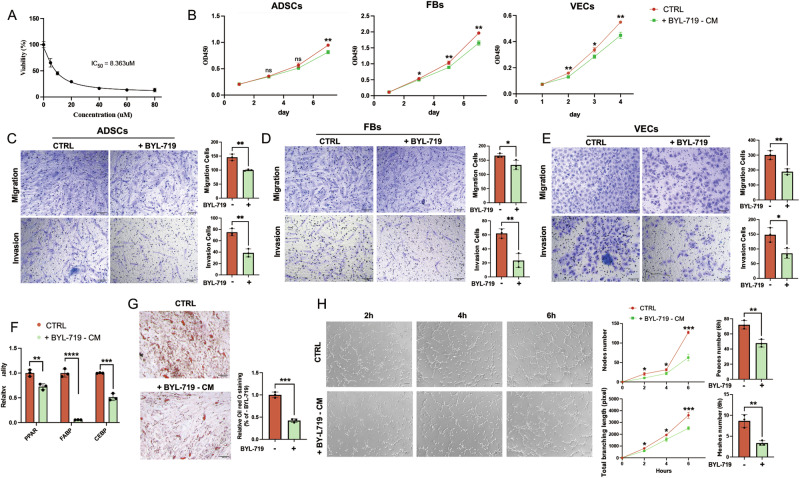
BYL-719 inhibits the paracrine effects of Mac-ADSCs. **A** Dose-response curve used to estimate the IC50 of BYL-719 in Mac-ADSCs. **B** Effect of BYL-719-treated Mac-ADSCs on proliferation of wild-type ADSCs, FBs, VECs. **C**–**E** Effect of BYL-719-treated Mac-ADSCs on migration and invasion capacities of (**C**) ADSCs, (**D**) FBs, and (**E**) VECs. **F**, **G** Adipogenic potential of wild-type ADSCs assessed by (**F**) mRNA levels of adipogenic markers and (**G**) Oil Red O staining after coculture with BYL719-treated Mac-ADSCs. **H** Tube formation capacity of VECs when cocultured with BYL-719-treated Mac-ADSCs. Scale bars: 100 μm. Data are presented as mean ± SD from three independent experiments. ns, not significant, *p < 0.05, **p < 0.01, ***p < 0.001, ****p < 0.0001 by unpaired *t* test.

BYL-719 (alpelisib), a selective PI3Kα inhibitor, has been identified as a promising therapeutic option for PROS [22, 23]. We further investigated whether BYL-719 could reverse the paracrine effects of Mac-ADSCs. A dose-response curve for BYL-719 was established and a half-maximal inhibitory concentration (IC50) of 8.363 μM (24 h) was selected for subsequent experiments (Fig. 5A). Western blot analysis demonstrated that BYL-719 can inhibited the phosphorylation levels of PI3K downstream effectors AKT and S6 (Figure S8A). Growth curve analyses revealed that BYL-719 significantly inhibited the effects of Mac-ADSCs on the proliferation of ADSCs, FBs, and VECs (Fig. 5B and Figure S8B). Additionally, BYL-719 markedly suppressed the effects of Mac-ADSCs on the migratory and invasive capacities of these cells, as demonstrated by Transwell assays (Fig. 5C–E and Figure S8C). Furthermore, BYL-719 markedly diminished the effect of Mac-ADSCs on the adipogenic capacity of ADSCs. The mRNA expression of adipogenesis-related genes (PPAR, FABP, and CEBP) was downregulated (Fig. 5F and Figure S8D). Consistent with these findings, Oil Red O staining revealed that BYL-719 abolished the paracrine-induced oil droplet accumulation in wild-type ADSCs (Fig. 5G). Finally, BYL-719 impaired the paracrine effects of Mac-ADSCs on angiogenesis in VECs, as evidenced by a reduction in the number of nodes, meshes, peaces and total branching length in tube formation assays (Fig. 5H and Figure S8E). These findings suggest that BYL-719 effectively suppresses the paracrine effects of Mac-ADSCs, suggesting a potential therapeutic strategy for treating macrodactyly and other PIK3CA-related overgrowth conditions.

## Discussion

**Fig. 6:**
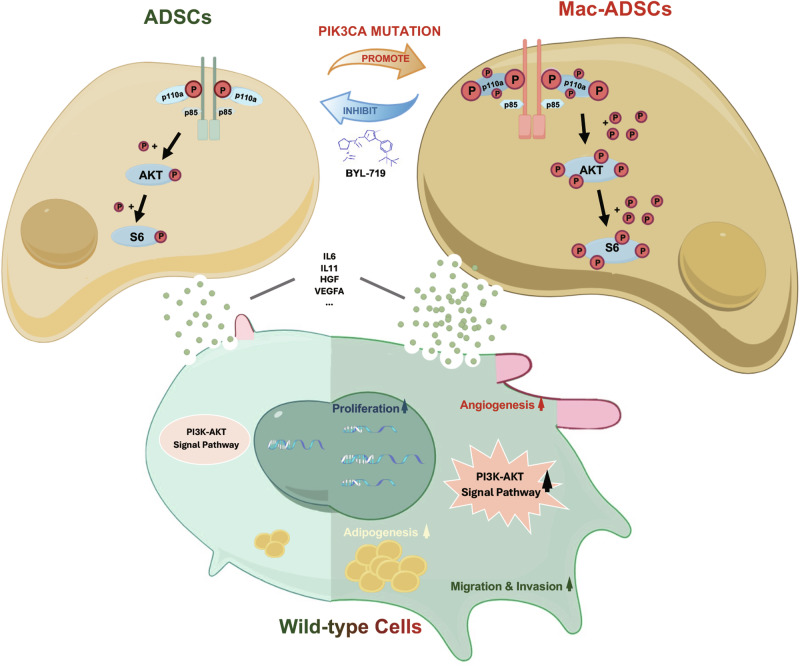
Summary of the paracrine effects of Mac-ADSCs. PIK3CA mutations activate the PI3K-AKT signaling pathway in ADSCs, transforming them into Mac-ADSCs and altering their cytokine secretion profile. The differentially secreted cytokines from Mac-ADSCs influence neighboring wild-type cells by activating their PI3K-AKT signaling pathway and enhancing their proliferation, migration, and invasion abilities. Additionally, these cytokines promote angiogenesis in VECs and adipogenesis in ADSCs. Notably, this paracrine effect can be reversed by the PIK3CA inhibitor BYL-719. This figure was created with Figdraw (https://www.figdraw.com/) and is permitted for publication.

Macrodactyly is a rare congenital limb deformity caused by gain-of-function mutations of PIK3CA. Although hyperplastic tissues are driven by these somatic changes, only a subset of cells harbor mutations under these mosaic conditions [24]. Previous research has focused primarily on the cell-autonomous effects of PIK3CA mutations [12, 13, 25]. However, the mosaic nature of these conditions highlights the need to explore the influence of intercellular communication. Kurek et al. suggested that the malformations are the result of paracrine signaling transformed from mutant to wild-type cells in CLOVES syndrome after analyzing the mutant allele frequency of different tissues [8]. Similarly, Chimenti et al. demonstrated that unaffected cardiomyocyte hypertrophy correlates with cardiomyopathy severity, which may be influenced by paracrine effects from neighboring affected myocytes [26]. However, how these mutated cells affect normal cells remains unknown. In this study, we provide the first experimental evidence demonstrating how PIK3CA mutations influence wild-type cells through paracrine signaling in macrodactyly. Our findings are summarized in Fig. 6.

Adipose tissue accumulation and dysregulation are prominent characteristics of macrodactyly [27]. Although previous research has demonstrated that Mac-ADSCs exhibit increased adipogenic potential [12], our findings expand upon this by demonstrating their paracrine effects on wild-type ADSCs. In addition, Mac-ADSCs promote the invasive and migratory capabilities of normal ADSCs, which may contribute to further hypertrophy of the affected limb. Among the cytokines we examined in the paracrine factors of Mac-ADSCs, HGF and VEGFA promoted the proliferation, migration and, adipogenic differentiation of normal ADSCs. Notably, IL-6 and IL-11 were found to inhibit the adipogenic ability of wild-type ADSCs. Both of them have been shown to activate the adenosine monophosphate-activated protein kinase (AMPK) signaling pathways and the signal transducer and activator of transcription 1 (STAT1) [28, 29], which are known to negatively regulate adipogenic differentiation. This suggests that pro-adipogenic factors like VEGFA, and possibly other unidentified factors, can override the adipogenesis-inhibiting signals from cytokines. Moreover, activation of the PI3K-AKT signaling pathway in wild-type ADSCs by Mac-ADSCs may potentially trigger an amplification cascade, which leads to a broader paracrine influence.

FBs play a key role in collagen deposition and tissue stiffness [30], both of which are prominent features of macrodactyly. ADSCs are generally believed to facilitate tissue regeneration by remodeling the behavior of FBs [31, 32]. Our study demonstrated that Mac-ADSCs promote the biological behavior of FBs, including their migration and collagen synthesis. The activation of FBs by Mac-ADSCs may contribute to the stiffening of hypertrophic tissues in macrodactyly, further exacerbating the deformity. We observed that the IL-11 and VEGFA secreted by Mac-ADSCs significantly impact the proliferation and migration of FBs. IL-11 has been reported to up-regulating a range of pro-fibrotic proteins and activating the JAK/STAT3 pathway in FBs, which promotes fibroblast activation and extracellular matrix production [33]. As a ubiquitous cytokine, VEGFA is known to activate various biological functions in multiple cell types and has been implicated in pathological processes such as keloid formation [34, 35]. Moreover, activated FBs can secrete cytokines to promote tissue growth and metastasis [36], indicating that activated FBs may influence the progression of macrodactyly. However, the precise role and underlying mechanisms of activated FBs in the disease progression remain to be fully explored, and future studies should aim to clarify their contribution to tissue remodeling in macrodactyly.

The growth of hyperplastic tissue depends on sustained vascularization, wherein VECs drive angiogenesis [37]. Our results showed that Mac-ADSCs promoted the proliferation, migration, and invasion of VECs, promoting angiogenesis in vitro. The neovascularization induced by Mac-ADSCs not only supplies the nutrients necessary for tissue proliferation but also contributes to the progression of lesions into healthy limb regions. Researches have demonstrated that ADSCs can enhance the proliferation and migration of vascular endothelial cells by secreting certain cellular factors, such as VEGFA and HGF [38]. Similarly, our findings confirm that VEGFA and HGF secreted by Mac-ADSCs promote the function of VECs. Additionally, IL-11 may further stimulate angiogenesis by activating FBs to secrete VEGFA [39], suggesting a complex interplay between Mac-ADSCs, FBs, and VECs in the progression of macrodactyly. However, notably, not all cases of macrodactyly exhibit vascular malformations [40]. We hypothesize that the well-developed adventitia of blood vessels may attenuate the paracrine influence of Mac-ADSCs on endothelial cells, potentially explaining the variability in vascular involvement among macrodactyly patients. In more severe cases, such as CLOVES syndrome or Klippel-Trenaunay syndrome, the absence of such protective mechanisms may lead to more extensive vascular malformations. Collectively, the above findings highlight a multifaceted regulatory network in macrodactyly. The increased activity of ADSCs further promotes the proliferation of adipose tissue in macrodactyly, directly contributing to the characteristic overgrowth of the affected digits. The proliferation of FBs and the elevated collagen synthesis can result in tissue fibrosis, contributing to the thickening and rigidity of the affected digits. Increased angiogenesis may facilitate the growth and expansion of adipose and fibrous tissues, exacerbating the morphological changes associated with macrodactyly. The interplay among adipogenesis, FB proliferation, collagen formation, and angiogenesis creates a microenvironment that supports the pathological features of macrodactyly, further driving its progression.

To elucidate the underlying mechanisms how PIK3CA-mutated ADSCs influence wild-type cells in macrodactyly, we performed RNA-sequencing and cytokine array analysis. Notably, alterations in the secretion of molecules belonging to the CCL and CXCL families were observed. These chemokines are small proteins with molecular weights ranging from 8 to 14 kDa, whose receptors are widely expressed on immune cells such as T cells, macrophages, dendritic cells, eosinophils, and microglia [41]. Under normal physiological conditions, chemokines are essential for orchestrating immune responses, guiding cellular trafficking during development, and maintaining tissue homeostasis by binding to class A G protein-coupled receptors [42]. Pathologically, elevated expression of chemokines may stimulate immune cell recruitment, thereby reshaping the microenvironment to support disease progression [43]. We also observed a general upregulation of the expression of IGFBP family molecules in the supernatant of Mac-ADSCs. These proteins primarily exert their functions by binding to IGFs and are closely associated with various biological processes, including cell growth, metabolism, and tumor progression [44]. However, we did not observe an elevation in IGF levels. Therefore, we hypothesize that the paracrine effects of IGFBPs secreted by Mac-ADSCs are independent of IGFs [45]. Additionally, we observed elevated secretion levels of several IL family members (IL-6, IL-7, and IL-11). Our functional validation of IL-6 and IL-11 revealed that IL-11 exerts a more significant influence on surrounding stromal cells in macrodactyly. This differential effect may be attributed to the distinct cellular distribution of their receptors, with IL-6R expressed predominantly on immune cells and IL-11RA mainly on stromal cells [33]. Moreover, IL-7 is essential for T cell development and activation [46]. We speculate that IL-6 and IL-7 may be more involved in shaping the immune microenvironment in macrodactyly. Furthermore, we observed alterations in other cytokines within the supernatant of Mac-ADSCs, suggesting their potential involvement in various cellular functions. For example, TIMP1, TIMP2, and uPAR, which play crucial roles in extracellular matrix remodeling and proteolysis [47, 48]. In the context of macrodactyly, these proteins may contribute to abnormal cell invasion and tissue remodeling. VEGFA, HGF, and angiogenin are known to promote angiogenesis under physiological conditions. The increased secretion of these factors by Mac-ADSCs likely contributes to increased angiogenesis, potentially leading to further tissue proliferation and overgrowth of macrodactyly. Furthermore, the reduced level of adiponectin secreted by Mac-ADSCs may lead to the presence of insulin resistance in PROS [22]. While our findings suggest that multiple cytokines are involved in these paracrine interactions, we validated only the effects of IL-6, IL-11, HGF, and VEGFA secreted by Mac-ADSCs on wild-type cells. Therefore, further studies are needed to determine the specific contributions of these factors to the activation of cellular behaviors across diverse cell populations.

Given the critical role of PIK3CA gain-of-function in mediating the paracrine effects of Mac-ADSCs, targeting the PI3K-AKT pathway may be a promising therapeutic strategy. BYL-719, a selective inhibitor of the p110α catalytic subunit of PI3K, was first applied in the treatment of estrogen receptor-positive breast cancer [49]. In PROS, Venot et al. [50] showed that BYL-719 substantially alleviates clinical symptoms and is more effective than rapamycin in treating CLOVES syndrome. This treatment has also been demonstrated to effectively alleviate lymphatic cystic malformations and severe metabolic dysfunction in patients with PIK3CA-related lymphatic and adipose malformations [22, 51]. Currently, BYL-719 is being evaluated in several clinical trials for PROS. A retrospective EPIK-P1 study (NCT04285723) demonstrated that BYL-719 reduced surgical necessity and alleviated symptoms in children with PROS [52, 53]. Moreover, a phase II trial (NCT04589650) is investigating its safety and efficacy in patients with PROS, including those with macrodactyly [54, 55]. Preliminary results from this and other studies have shown promising outcomes of PIK3CA inhibitor therapy, with significant reductions in overgrowth and improvements in quality of life for many patients. Building upon existing clinical observations, this study offers new insights into the mechanism of action of BYL-719 in macrodactyly treatment. We demonstrated that BYL-719 not only targets PIK3CA-mutated cells, but also reduces their paracrine influence on surrounding normal cells, which may offer a more comprehensive therapeutic approach than previously recognized.

In summary, this study represents the first systematic investigation into the paracrine functions of PIK3CA-mutated ADSCs in the context of congenital disease. Our findings demonstrate that Mac-ADSCs secrete a range of cytokines that activate the PI3K-AKT pathway in adjacent wild-type cells, driving mutant-like phenotypes. These findings highlight the importance of paracrine signaling in the pathogenesis of macrodactyly and provide evidence supporting the hypothesis that paracrine effects can induce abnormalities in normal cells in other mosaic mutation disorders. Meanwhile, targeting the paracrine factors of Mac-ADSCs with neutralizing antibodies could represent a potential therapeutic strategy to inhibit progression in macrodactyly. Recognizing that macrodactyly is a characteristic feature of various PROS conditions, which are also defined by mesodermal dysplasias, our macrodactyly-centric study provides relevant insights into the pathogenic mechanisms underlying a significant portion of the PROS spectrum. In the future, systematic comparative studies of histological features across different PROS entities would be highly valuable to further elucidate the specific pathological mechanisms underlying macrodactyly and its relationship to the broader PROS spectrum. In addition to its implications for macrodactyly, this study offers insights that could impact the broader fields of cancer biology and regenerative medicine. Understanding how mutant cells influence their microenvironment through paracrine signaling could offer new insights into tumor progression and metastasis in PIK3CA-mutated cancers. Furthermore, the paracrine mechanisms elucidated here may be relevant to other PIK3CA-related overgrowth syndromes and could inform strategies for promoting tissue regeneration and wound healing in various contexts.

## Supplementary information

Supplemental Figure 1

Supplemental Figure 2

Supplemental Figure 3

Supplemental Figure 4

Supplemental Figure 5

Supplemental Figure 6

Supplemental Figure 7

Supplemental Figure 8

Supplemental Figure legends

Supplemental Table 1

Supplemental Table 2

Supplemental Table 3

Supplemental Table 4

Raw western

## Data Availability

The data supporting the findings of this study are available from the corresponding author upon reasonable request.
