# Supplementary figures and images for: Activating PIK3CA mutations in adipose-derived stem cells drive mutant-like phenotypes of wild-type cells in macrodactyly
Source: Cell Death Dis. 2025 Jul 1;16(1):477. doi: 10.1038/s41419-025-07795-7 (PMC12217521; doi:10.1038/s41419-025-07795-7)

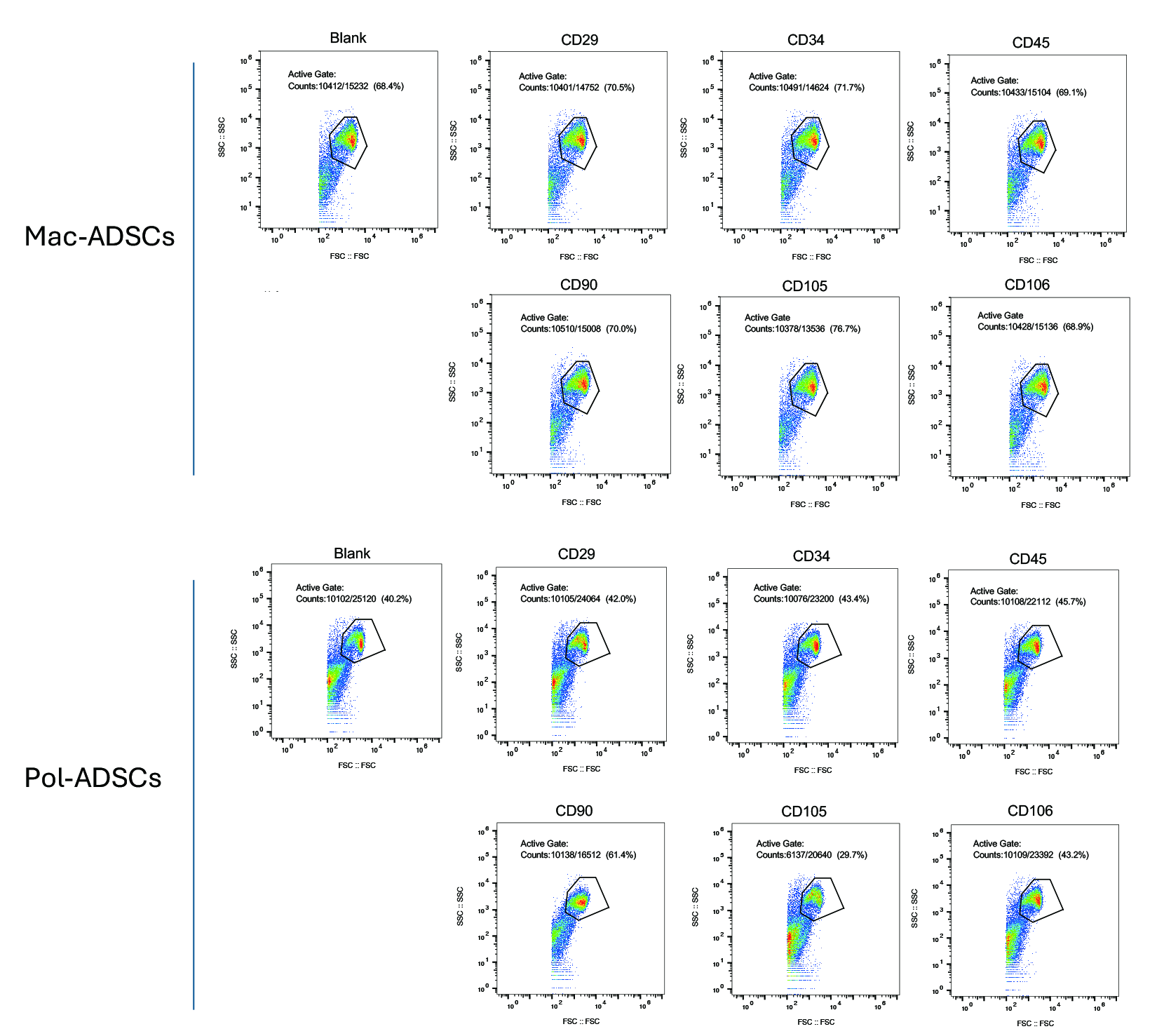

Supplement: Supplementary file 1 — Supplemental Figure 1 [file 41419_2025_7795_MOESM1_ESM.tif]

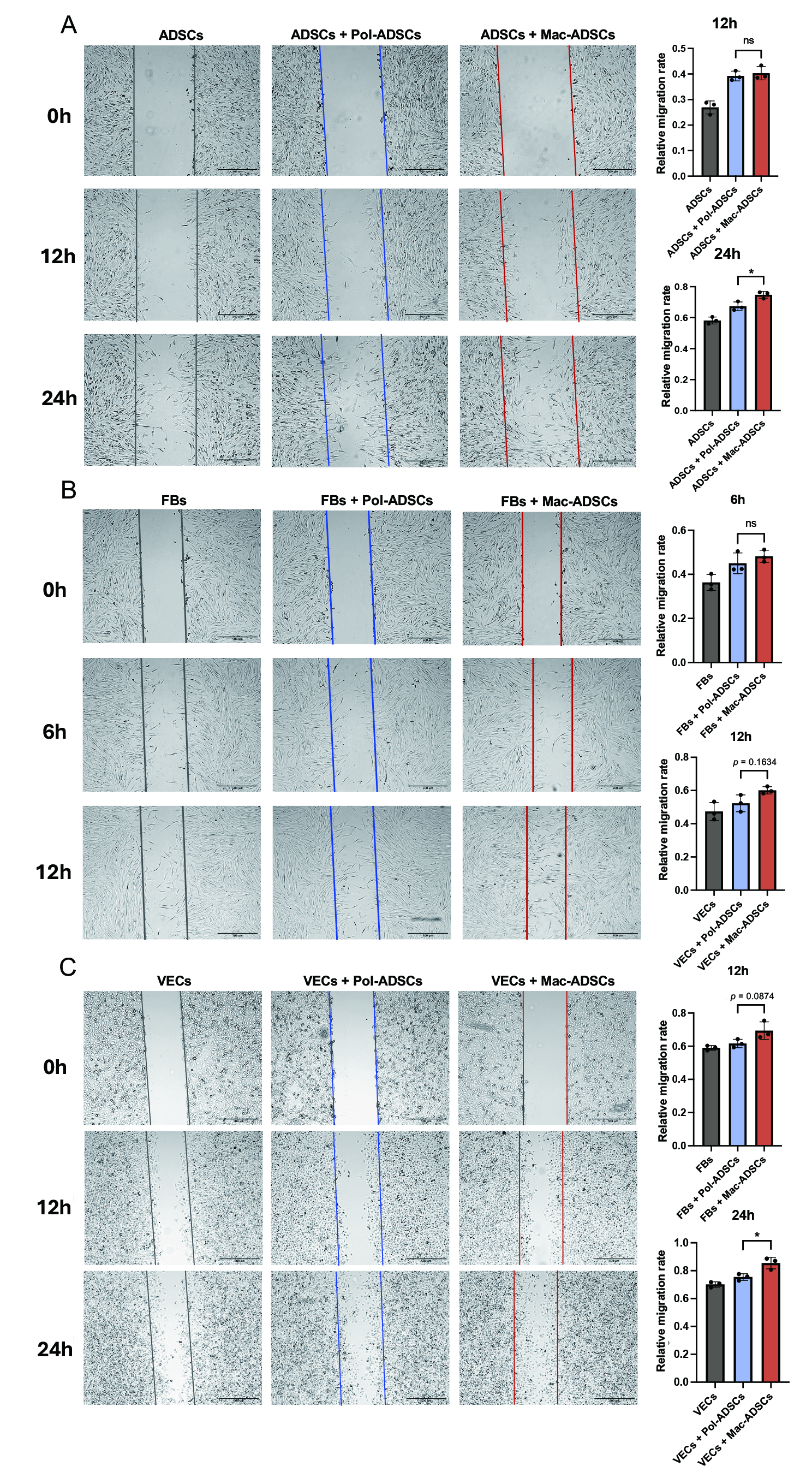

Supplement: Supplementary file 2 — Supplemental Figure 2 [file 41419_2025_7795_MOESM2_ESM.tif]

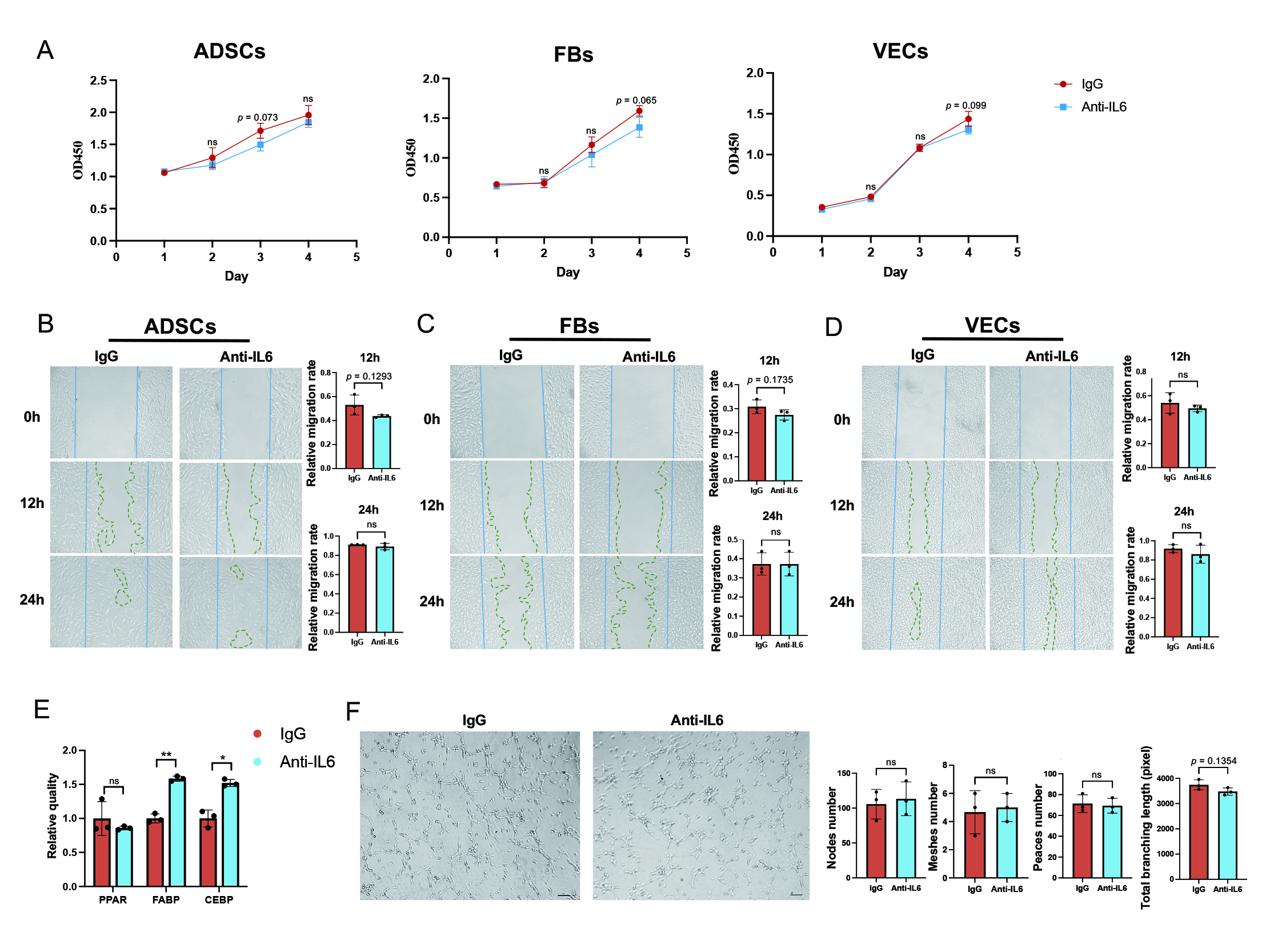

Supplement: Supplementary file 3 — Supplemental Figure 3 [file 41419_2025_7795_MOESM3_ESM.tif]

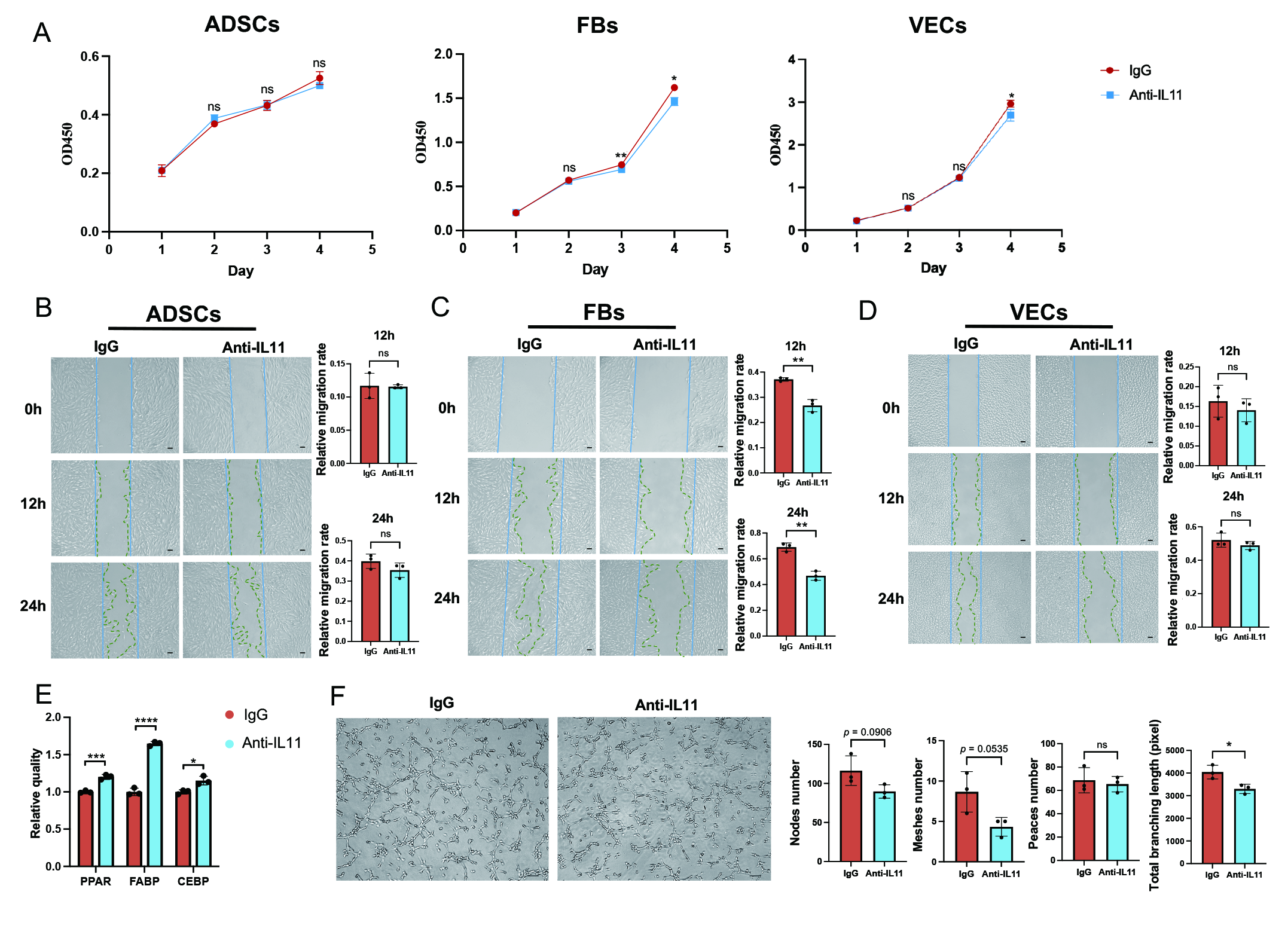

Supplement: Supplementary file 4 — Supplemental Figure 4 [file 41419_2025_7795_MOESM4_ESM.tif]

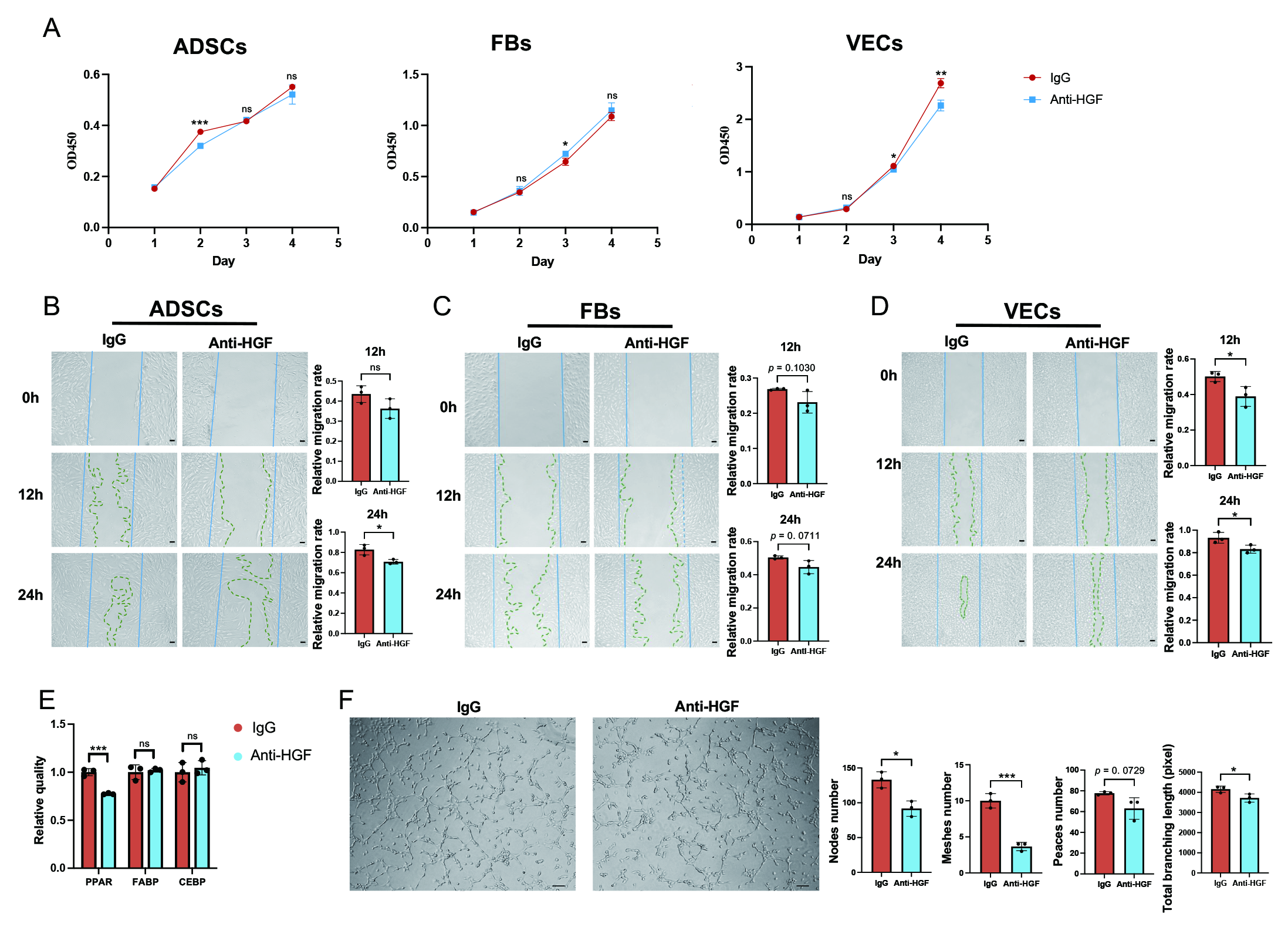

Supplement: Supplementary file 5 — Supplemental Figure 5 [file 41419_2025_7795_MOESM5_ESM.tif]

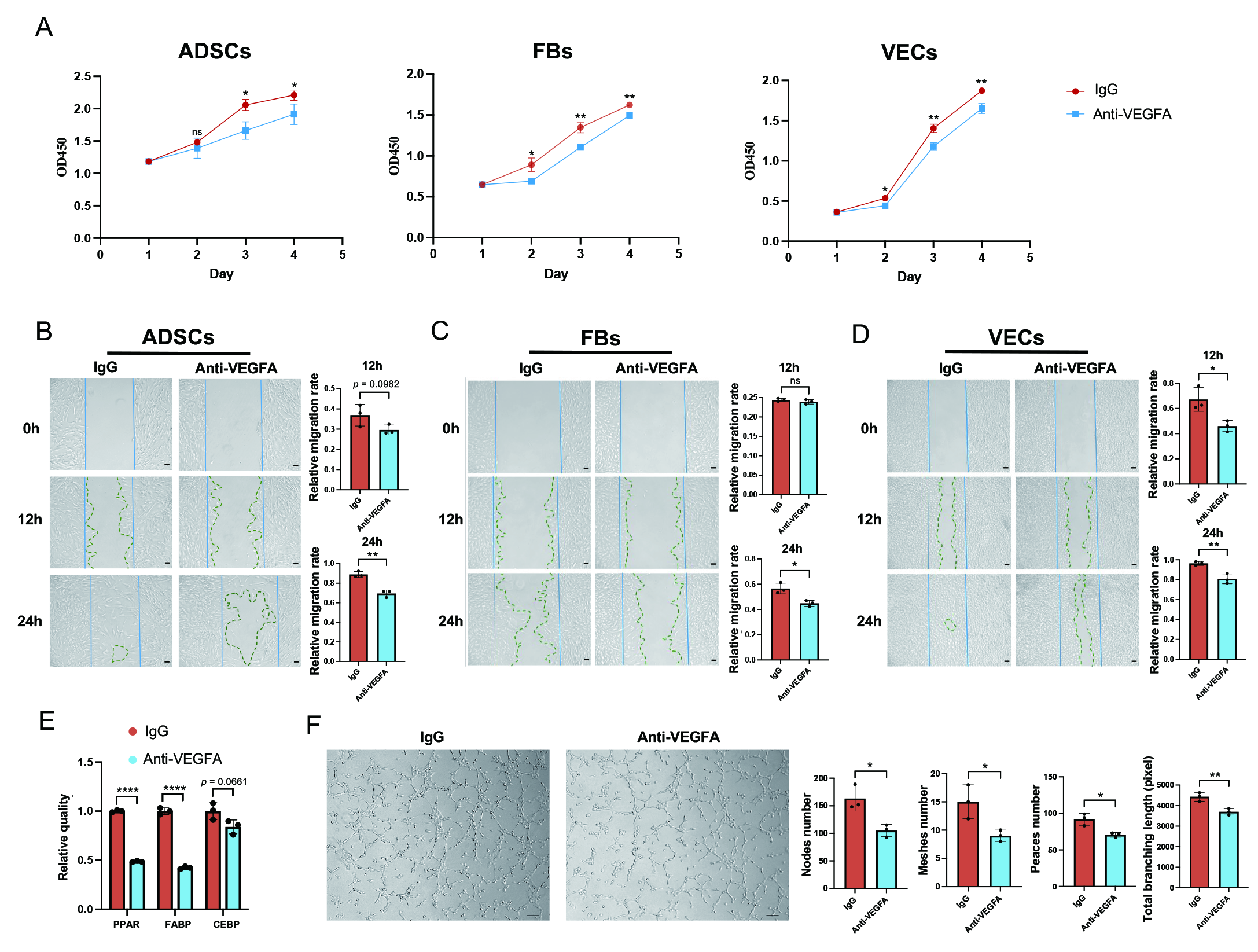

Supplement: Supplementary file 6 — Supplemental Figure 6 [file 41419_2025_7795_MOESM6_ESM.tif]

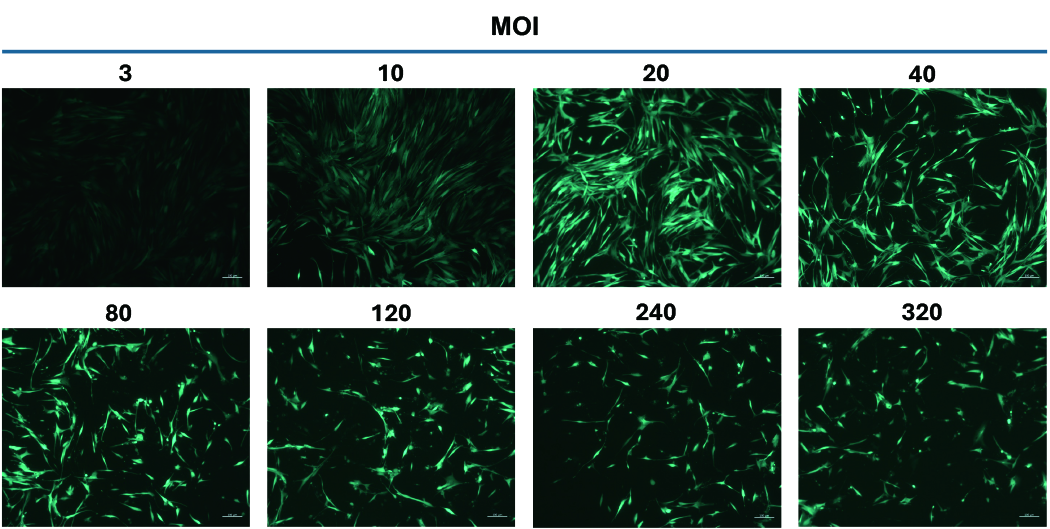

Supplement: Supplementary file 7 — Supplemental Figure 7 [file 41419_2025_7795_MOESM7_ESM.tif]

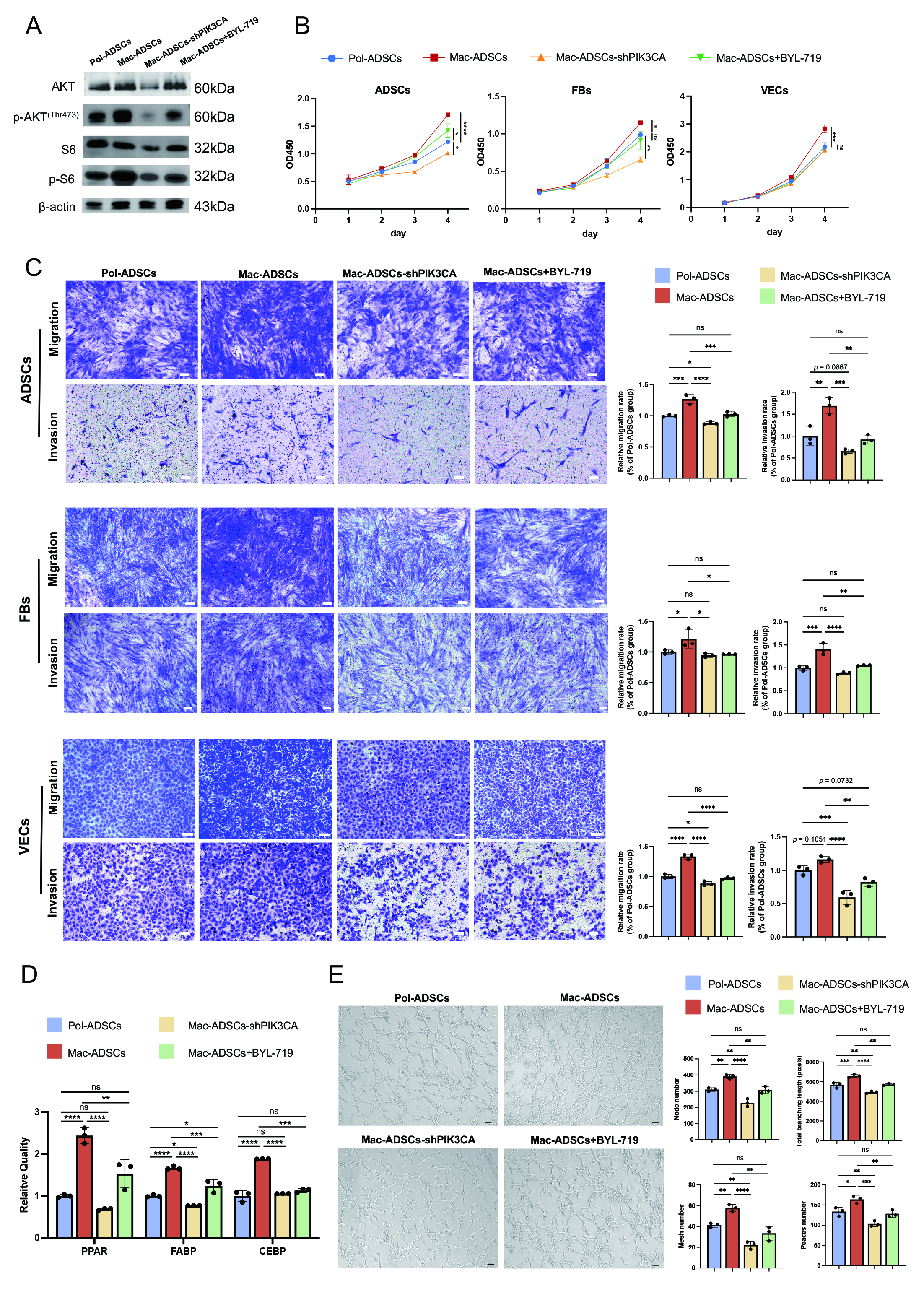

Supplement: Supplementary file 8 — Supplemental Figure 8 [file 41419_2025_7795_MOESM8_ESM.tif]

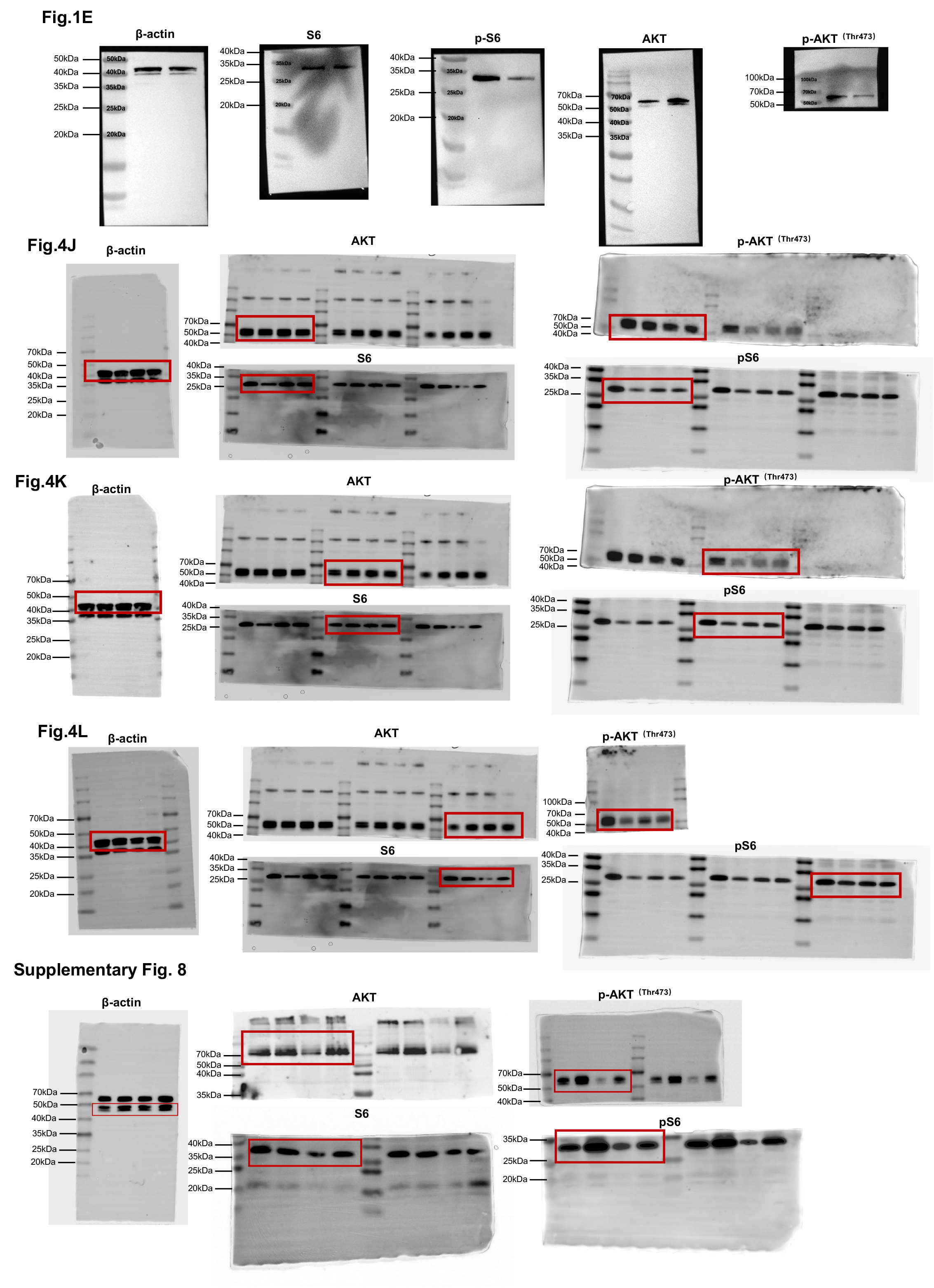

Supplement: Supplementary file 14 — Raw western [file 41419_2025_7795_MOESM14_ESM.tif]
