# Supplementary material for: Activating PIK3CA mutations in adipose-derived stem cells drive mutant-like phenotypes of wild-type cells in macrodactyly
Source: Cell Death Dis. 2025 Jul 1;16(1):477. doi: 10.1038/s41419-025-07795-7 (PMC12217521; doi:10.1038/s41419-025-07795-7)
Supplement: Supplementary file 9 — Supplemental Figure legends [file 41419_2025_7795_MOESM9_ESM.docx]

**Supplemental Figure legends:**

**Fig. S1 Gating strategy for flow cytometric analysis of ADSCs.** Forward scatter (FSC) and side scatter (SSC) plots showing initial gating to exclude debris and select the main cell population. Numbers and percentages of cells in the active gate for each marker (CD29, CD34, CD45, CD90, CD105, CD106) are indicated within the respective plots.

**Fig. S2 Mac-ADSCs enhance migration of wild-type cells.** (A-C) Representative images and quantification of scratch assays showing the effect of Mac-ADSCs on the migration of (A) ADSCs, (B) FBs, and (C) VECs. Data are presented as mean ± SD from three independent experiments. ns, not significant, **p* < 0.05 by ANOVA analysis.

**Fig. S3** **Effects of IL-6 neutralization on Mac-ADSC-mediated paracrine signaling.** (A) Proliferation of ADSCs, FBs, and VECs when cultured in Mac-ADSCs conditioned medium (CM) with or without IL-6 neutralizing antibody. (B-D) Migration capacities assessed by scratch wound assay of (B) ADSCs, (C) FBs, and (D) VECs when cultured in Mac-ADSCs-CM with or without IL-6 neutralizing antibody. (E) Adipogenic differentiation potential of ADSCs cultured in Mac-ADSCs-CM with or without IL-6 neutralizing antibody. (F) Tube formation capacity of VECs when cultured in Mac-ADSCs-CM with or without IL-6 neutralizing antibody. Data are presented as mean ± SD from three independent experiments. ns, not significant, **p* < 0.05, ***p* < 0.001 by unpaired t test.

**Fig. S4 Effects of IL-11 neutralization on Mac-ADSC-mediated paracrine signaling.** (A) Proliferation of ADSCs, FBs, and VECs when cultured in Mac-ADSCs conditioned medium (CM) with or without IL-11 neutralizing antibody. (B-D) Migration capacities assessed by scratch wound assay of (B) ADSCs, (C) FBs, and (D) VECs when cultured in Mac-ADSCs-CM with or without IL-11 neutralizing antibody. (E) Adipogenic differentiation potential of ADSCs when cultured in Mac-ADSCs-CM with or without IL-11 neutralizing antibody. (F) Tube formation capacity of VECs when cultured in Mac-ADSCs-CM with or without IL-11 neutralizing antibody. Data are presented as mean ± SD from three independent experiments. ns, not significant, **p* < 0.05, ***p* < 0.01, ****p* < 0.001, *****p* < 0.001 by unpaired t test.

**Fig. S5 Effects of HGF neutralization on Mac-ADSC-mediated paracrine signaling.** (A) Proliferation of ADSCs, FBs, and VECs when cultured in Mac-ADSCs conditioned medium (CM) with or without HGF neutralizing antibody. (B-D) Migration capacities assessed by scratch wound assay of (B) ADSCs, (C) FBs, and (D) VECs when cultured in Mac-ADSCs-CM with or without HGF neutralizing antibody. (E) Adipogenic differentiation potential of ADSCs when cultured in Mac-ADSCs-CM with or without HGF neutralizing antibody. (F) Tube formation capacity of VECs when cultured in Mac-ADSCs-CM with or without HGF neutralizing antibody. Data are presented as mean ± SD from three independent experiments. ns, not significant, **p* < 0.05, ***p* < 0.01, ****p* < 0.001 by unpaired t test.

**Fig. S6 Effects of VEGFA neutralization on Mac-ADSC-mediated paracrine signaling.** (A) Proliferation of ADSCs, FBs, and VECs when cultured in Mac-ADSCs conditioned medium (CM) with or without VEGFA neutralizing antibody. (B-D) Migration capacities assessed by scratch wound assay of (B) ADSCs, (C) FBs, and (D) VECs when cultured in Mac-ADSCs-CM with or without VEGFA neutralizing antibody. (E) Adipogenic differentiation potential of ADSCs when cultured in Mac-ADSCs-CM with or without VEGFA neutralizing antibody. (F) Tube formation capacity of VECs when cultured in Mac-ADSCs-CM with or without VEGFA neutralizing antibody. Data are presented as mean ± SD from three independent experiments. ns, not significant, **p* < 0.05, ***p* < 0.01, ****p* < 0.001, *****p* < 0.0001 by unpaired t test.

**Fig.S7 Visualization of lentiviral infection efficiency in Mac-ADSCs.** Fluorescence images showing GFP expression at different multiplicity of infection (MOI) values.

**Fig. S8 Comparison of paracrine functions between Pol-ADSCs and differently treated Mac-ADSCs.** (A) Activation status of PIK3CA downstream signaling pathways. (B) Proliferation of ADSCs, FBs, and VECs when cultured with conditioned medium (CM) from different ADSCs. (C) Migration and invasion capacities of ADSCs, FBs, and VECs when cocultured with different ADSCs. (D) mRNA expression levels of adipogenic markers when cultured with CM from different ADSCs. (E) Tube formation capacity of VECs when cultured with CM from different ADSCs. Scale bars: 100 μm. Data are presented as the mean ± SD from three independent experiments. *p < 0.05, **p < 0.01, ***p < 0.001, ****p < 0.0001 by ANOVA analysis.
