# Supplementary material for: Activating PIK3CA mutations in adipose-derived stem cells drive mutant-like phenotypes of wild-type cells in macrodactyly
Source: Cell Death Dis. 2025 Jul 1;16(1):477. doi: 10.1038/s41419-025-07795-7 (PMC12217521; doi:10.1038/s41419-025-07795-7)
Supplement: Supplementary file 10 — Supplemental Table 1 [file 41419_2025_7795_MOESM10_ESM.docx]

| **Table S1. Clinical information of patients** | | | | | | | |
| --- | --- | --- | --- | --- | --- | --- | --- |
| **Case No.** | **Sex** | **Age** | **Diagnosis** | **Location** | **Surgical procedure** | **Specimen** | **Application** |
| 1 | Male | 1y | Macrodactyly | Left hand | Surgical de-bulking | Adipose tissue of macrodactyly | Sanger sequencing, histological stain, cytokine array analysis, ADSCs isolation |
| 2 | Female | 1y | Macrodactyly | Right hand | Surgical de-bulking | Adipose tissue of macrodactyly | Sanger sequencing, histological stain, ADSCs isolation |
| 3 | Male | 2y | Macrodactyly | Left hand | Surgical de-bulking | Adipose tissue of macrodactyly | Sanger sequencing, histological stain, ADSCs isolation |
| 4 | Male | 3y | Macrodactyly | Right feet | Surgical de-bulking | Adipose tissue of macrodactyly | Sanger sequencing, ADSCs isolation |
| 5 | Male | 8m | Polydactyly | Right hand | Polydactyly resection | Subcutaneous adipose tissue of polydactyly | Sanger sequencing, histological stain, cytokine array analysis, ADSCs isolation |
| 6 | Female | 10m | Polydactyly | Left hand | Polydactyly resection | Subcutaneous adipose tissue of polydactyly | Sanger sequencing, histological stain, ADSCs isolation |
| 7 | Female | 2y | Polydactyly | Right hand | Polydactyly resection | Subcutaneous adipose tissue of polydactyly | Histological stain, ADSCs isolation |
| 8 | Male | 10m | Polydactyly | Left hand | Polydactyly resection | Subcutaneous adipose tissue of polydactyly | ADSCs isolation |
| 9 | Male | 1y | Polydactyly | Right hand | Polydactyly resection | Subcutaneous adipose tissue of polydactyly | ADSCs isolation |
| 10 | Male | 1.5y | Polydactyly | Left hand | Polydactyly resection | Subcutaneous adipose tissue of polydactyly | ADSCs isolation |
