# Supplementary material for: Activating PIK3CA mutations in adipose-derived stem cells drive mutant-like phenotypes of wild-type cells in macrodactyly
Source: Cell Death Dis. 2025 Jul 1;16(1):477. doi: 10.1038/s41419-025-07795-7 (PMC12217521; doi:10.1038/s41419-025-07795-7)
Supplement: Supplementary file 11 — Supplemental Table 2 [file 41419_2025_7795_MOESM11_ESM.docx]

| Antibodies | Source | Identifier | Dilution |
| --- | --- | --- | --- |
| Anti-CD29 | Biolegend | Cat No. 303015 | FC 5μl/Test |
| Anti-CD34 | Biolegend | Cat No. 343603 | FC 5μl/Test |
| Anti-CD45 | Biolegend | Cat No. 304006 | FC 5μl/Test |
| Anti-CD90 | Biolegend | Cat No. 328107 | FC 5μl/Test |
| Anti-CD105 | Biolegend | Cat No. 800505 | FC 5μl/Test |
| Anti-CD106 | eBioscience | Cat No.53-1069-42 | FC 5μl/Test |
| Anti-IL6 | Proteintech | Cat No.69001-1-lg | Neutralising 10μg/ml |
| Anti-IL11 | R&D SYSTEMS | Cat No. MAB218 | Neutralising 10μg/ml |
| Anti-HGF (Rilotumumab) | MedChemExpress | Cat No. HY-P99217 | Neutralising 10ug/ml |
| Anti-VEGFA  (Bevacizumab) | MedChemExpress | Cat No. HY-P9906 | Neutralising 5ug/ml |
| Anti-PIK3CA | Cell Signaling Technology | Cat No. 4249 | WB 1:1000 |
| Anti-AKT | Cell Signaling Technology | Cat No. 4691 | WB 1:1000 |
| Anti-pAKT | Cell Signaling Technology | Cat No. 13038 | WB 1:1000 |
| Anti-S6 | Cell Signaling Technology | Cat No. 2317 | WB 1:2000 |
| Anti-pS6 | Cell Signaling Technology | Cat No. 4858 | WB 1:2000 |
| Anti-β-actin | Cell Signaling Technology | Cat No. 4967 | WB 1:5000 |
| Anti‒rabbit IgG, HRP‒linked Antibody | Cell Signaling Technology | Cat No. 7074S | WB 1:2500 |
| Anti‒mouse IgG, HRP‒linked Antibody | Cell Signaling Technology | Cat No. 7076 | WB 1:2500 |

**Table S2. Antibodies used in experiments**
