# Supplementary material for: Activating PIK3CA mutations in adipose-derived stem cells drive mutant-like phenotypes of wild-type cells in macrodactyly
Source: Cell Death Dis. 2025 Jul 1;16(1):477. doi: 10.1038/s41419-025-07795-7 (PMC12217521; doi:10.1038/s41419-025-07795-7)
Supplement: Supplementary file 12 — Supplemental Table 3 [file 41419_2025_7795_MOESM12_ESM.docx]

| **Table S3. Prime sequence for quantitative RT-PCR** | |
| --- | --- |
| **Genes** | **Primer sequence** |
| PPAR γ | F:TGTCTCATAATGCCATCAGGTTTG |
|  | R:GATAACGAATGGTGATTTGTCTGTT |
| C/EBP α | F:GGACCCTCAGCCTTGTTTGT |
|  | R:TGGTGGTTTAGCAGAGACGC |
| FABP4 | F:ACCAGGAAAGTGGCTGGCAT |
|  | R:CAGGTCAACGTCCCTTGGCT |
| PIK3CA | F:TCGCCTCATAGCAGAGCAAT |
|  | R:AGGACAACAACATGCTCCGA |
| COL3A1 | F:CCATCAGGACTAATGAGGCTTTC |
|  | R:CCCCGTATTATGGAGATGAACC |
| α-SMA | F:CGGCTTCATCGTATTCCTGTT |
|  | R:CAATGTCCTATCAGGGGGCAC |
| GAPDH | F:GGAGCGAGATCCCTCCAAAAT |
|  | R:GGCTGTTGTCATACTTCTCATGG |
|  |  |
|  |  |
